# Preparation of Novel Mesoporous Silica Using a Self-Assembled Graphene Oxide Template

**DOI:** 10.1038/s41598-020-63017-4

**Published:** 2020-04-10

**Authors:** Kyeong-Won Park, Oh-Yun Kwon

**Affiliations:** 1 Department of Chemistry and Faculty of General Education, Gyeongsang National University, Jinju, 52828 Republic of Korea; 2 Department of Chemical and Biomolecular Engineering, Chonnam National University, Yosu, Chonnam 59626 Republic of Korea

**Keywords:** Synthesis and processing, Synthesis and processing

## Abstract

Novel mesoporous silicas rolled with silica sheets with 2D regular spacing were prepared using a self-assembled graphene oxide (GO) template formed by mixing GO with Pluronic123 (P123). Self-assembled GO templated mesoporous silicas (SGT-PMS) showed well-developed X-ray diffraction peaks with *d*-spacings of 9.8–10.8 nm depending on the amount of GO, indicating mesoporous structures. The specific surface areas increased from 603.8 to 861.2 m^2^g^−1^ on adding GO. The pore size distribution was in the range 5.1–5.8 nm and pore volume in the range 0.80–0.99 m^3^g^−1^. The SEM images of SGT-PMS showed irregular elliptical particles with various sizes. TEM images showed that the cross section of SGT-PMS particles comprises a roll of silica sheets with 2D regular spacing. The pore walls of SGT-PMS are firmer and thicker than those for PMS without GO as indicated by the corresponding intensities of Q^3^ and Q^4^ signals. These results were explained well by the self-assembled GO templating mechanism.

## Introduction

Periodic mesoporous silica (PMS) has attracted great interest in fields such as separation, adsorption, catalysis, optics, and biomedicine due to excellent properties such as high porosities, large specific surface areas, low densities, tunable and large pore sizes, surface hydrophilicity, and biocompatibility^1–10^.

Zeolites, which are microporous materials with three-dimensional aluminosilicate skeleton structures and constant pore sizes of ≤1 nm, are also referred to as molecular sieves^11,12^.

They were also expected to be suitable catalyst carriers for the catalytic cracking process of the heavy oil. However, they had to improve the limited pore size for practical use. To overcome this limitation, researchers in Mobil synthesized MCM-41^13^ and MCM-48^14^, which drastically increased studies on the synthesis and application of PMS in nanochemical fields as adsorbents and catalyst carriers^14–19^.

PMS related materials such as MCM-41^13^, SBA-15^1^, and FSM-16^20,21^ are typically synthesized using a surfactant micelle-template. Their pore characteristics were also altered by changing the structure and morphology of micelles, which was achieved by adjusting the surfactant type, molecular structure, concentration, and additives^17,19,22,23^.

In particular, MCM-41 related materials synthesized using a cationic surfactant micelle-template were expected to be highly suitable as catalyst carriers because of their regular and uniform pore structure. However, their pore structures collapsed easily under hydrothermal conditions or at temperatures higher than 700 °C because of their thin pore walls. SBA-15 has also attracted attention as an adsorbent and catalyst carrier in nanochemical fields because it is thermally stable and has excellent pore characteristics like large surface areas and regular pore sizes of ~5 nm^1^. We also reported that PMS with stable pore structure could be prepared using a nonionic surfactant micelle-template^23^. Dipole interactions occur between the ethylene oxide chain in the nonionic surfactant and metal hydroxide ions^18,23–27^. When the metal hydroxide ions are stabilized and solidified by these interactions, the pore walls of the PMS are expected to be thicker than the ethylene oxide chain length. The formation of a thick pore wall results in a large specific surface area and an excellent thermal stability.

In recent years, the synthesis of PMS using graphene oxide (GO) as a template^28,29^ and graphene-PMS hybrids such as sandwich-type nanocomposites of GO and PMS^30–32^ have attracted interest. The extraordinary properties of graphene such as single atomic layer carbon structure, excellent electrical conductivity, mechanical stiffness, thermal conductivity, and chemical reactivity^33–38^ have been combined with other functionalities by forming nanocomposites with metal and metal oxides^28,39–42^.

In this study, novel PMS rolled with 2D silica sheets are successfully synthesized using a self-assembled GO template (SGT). When GO sheets are added to a Pluronic123 (P123) solution, hydrophilic groups of P123 molecules are adsorbed on the surface of GO sheets by interacting with π-electrons of GO sheet, resulting in more hydrophobic GO sheets. Such hydrophobic GO sheets form self-assembled GO aggregates, which form a roll stacked with GO sheets due to the neutral interaction between surface alkyl groups. Tetraethyl orthosilicate (TEOS) is easily introduced into the hydrophobic galleries between GO sheets thereby composing self-assembled GO aggregates. The gelation of TEOS in the gallery produces rolls sandwiched with silica sheets between GO sheets. Their calcination results in a roll of two-dimensional silica sheets with very regular spacing.

## Materials and methods

### Preparation of GO

GO was prepared according to the modified Hummer’s method^29,43,44^. Briefly, in a 2-L three-necked round-bottom flask, commercial graphite powder (Aldrich, 5 g) and NaNO_3_ (3.75 g) were added to concentrated H_2_SO_4_ (350 mL). This mixture was stirred in an ice-water bath, and 20 g of KMnO_4_ was slowly added over 1 h, followed by continuous stirring for 2 h in the ice-water bath. After the mixture was stirred vigorously for 2 days at room temperature, 700 mL of a 5 wt.% H_2_SO_4_ aqueous solution was added over 1 h with stirring at a constant temperature of 98 °C. The resultant mixture was further stirred for 2 h at 98 °C. After the temperature was reduced to 60 °C, 15 mL of H_2_O_2_ (30 wt.% aqueous solution) was added, and the mixture was stirred for 2 h at room temperature. To remove extraneous oxidation products and other inorganic impurities, the resultant mixture was purified by repeating the following procedure 20 times: centrifugation, removal of the supernatant liquid, dispersion of the solid using vigorous stirring, and ultrasonication for 1 h at a power of 150 W. The resultant solid was recovered by centrifugation, washed with deionized water and ethanol (EtOH) until H^+^ was removed, and then dried in air at 40 °C.

### Synthesis of SGT-PMS-x

The weight ratios of the reactant solutions are listed in Table 1. Briefly, 1.0 g of P123 (*M*_av_ = 5800, Aldrich) was dissolved in a mixture of 30 mL of 2-N HCl and 8.0 mL of distilled water at 38 °C. Then a certain amount of as-prepared GO (weighing 0, 1.5, 2.2 or 3.0 mg) was added and homogeneously dispersed under sonication. For each solution, 2.15 g of TEOS (Aldrich) was added with vigorous stirring for 5 min, resulting in gel type precipitation. Here, SGT was also prepared by homogeneously dispersing under sonication after adding 3.0 mg of as-prepared GO to 30 ml of above P123 solution without TEOS. The resultant solutions were aged for 5 h at 80 °C. As-synthesized solid samples were recovered by filtration and washed with deionized water, and then dried for 3 h at 80 °C. The dried samples were labelled as SGT and as-synthesized SGT-PMS-x (x = 1.5, 2.2, 3.0 mg) and x represented the different weights (mg) of GO added. Finally, dried powders of as-synthesized SGT-PMS-x were calcined in a furnace for 5 h at 600 °C in air, which removed the P123 and GO templates. The final samples were also labelled as SGT-PMS-x (x = 1.5, 2.2, 3.0) and x represented different weights (mg) of GO added. For comparison, the ordered mesoporous silica prepared without GO was labelled as PMS-0.

**Table 1:** Mass ratios of the reactant solutions for the preparation of SGT, PMS-0 and SGT-PMS-x.

Sample	GO (mg)	P123 (g)	TEOS (g)
SGT	3	1	0
PMS-0	0	1	2.15
SGT-PMS-1.5	1.5	1	2.15
SGT-PMS-2.2	2.2	1	2.15
SGT-PMS-3.0	3	1	2.15

### Characterization

The powder X-ray diffraction (XRD) patterns of the samples were recorded on a Bruker D8-Advance X-ray powder diffractometer using Cu Kα radiation (λ = 0.1542 nm) with scattering angles (2θ) of 1°–10°, operating at 40 keV, with a cathode current of 20 mA. Fourier transform infrared (FT-IR) were recorded using Attenuated Total Reflectance (ATR) sampling accessory on the Nicolet iS50 FT-IR spectrometer. X-ray photoelectron spectra (XPS) were recorded using a VersaProbe, Ulvac-PHI, with Al *Ka* excitation radiation (hv = 1486.6 eV). The pressure in the analyser was maintained at approximately 6.7 × 10^−7^ Pa. XPS data was processed using a DS 300 data system.

Scanning electron micrographs were obtained using a JEOL JSM-840A scanning electron microscope (SEM). The transmission electron micrographs (TEM) were obtained with a JEOL JEM-200 CX transmission electron microscope operated at 200 kV, using a thin-section technique. The powder samples were embedded in epoxy resin and then sectioned with ultra-microtome. Atomic force microscopy (AFM) images were obtained using an AutoProbe CP/MT scanning probe microscope (XE-100(PSIA)). Imaging was performed in non-contact mode using a V-shaped Ultralever probe B (Park Scientific Instruments, B-doped Si with a frequency ƒ_c_ = 78.6 kHz, spring constant *k* = 2.0–3.8 N m^−1^, and nominal tip radius = 10 nm). All images were collected under ambient conditions at 50% relative humidity and 23 °C with a scanning raster rate of 1 Hz. Samples for AFM were prepared by depositing dispersions of GO in EtOH on a freshly cleaved mica surface (Ted Pella Inc. Prod No. 50) and allowing them to air-dry.

Solid-state ^29^Si MAS NMR spectra was recorded on a Bruker CXP-100 spectrometer at a resonance frequency of 19.89 MHz with a 45° pulse and a recycle delay of 7 s. Raman spectra were obtained using a Jobin Yvon/Horiba LabRAM spectrometer equipped with an integral microscope (Olympus BX 41). A 514.5-nm Ar-laser was used as an excitation source. Samples were sonicated in EtOH and three drops were placed on a glass slide for observation. The samples were viewed using green and red laser apparatuses with maximum magnifications of 50× and 100×, respectively. N_2_ adsorption isotherms were obtained at 77 K using a nitrogen sorption instrument (Micromeritics ASAP 2020). Pore size distributions were calculated by the Barrett–Joyner–Halenda (BJH) method using the adsorption branches of the isotherms.

## Results and discussion

**Figure 1:**
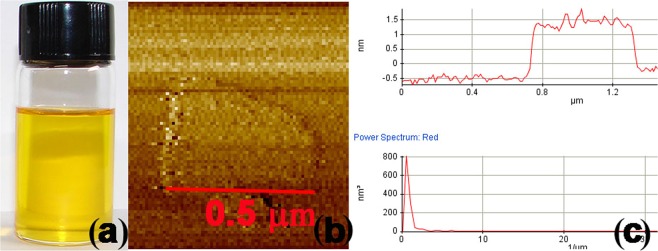
(**a**) Photograph of the GO solution, (**b**) AFM image of a GO sheet, (**c**) topography and height profile of a GO sheet.

Figure 1(a) shows a photograph of the GO solution, showing well-formed GO as indicated by the yellow colour^29^; Fig. 1(b,c) show AFM images indicating GO particles with a thickness of ~1 nm and length of ~0.5 μm, corresponding with previous reports^45,46^. However, the GO prepared in this work comprises single or multiple sheets, because AFM images can be discriminated well for the single GO sheets.

**Figure 2:**
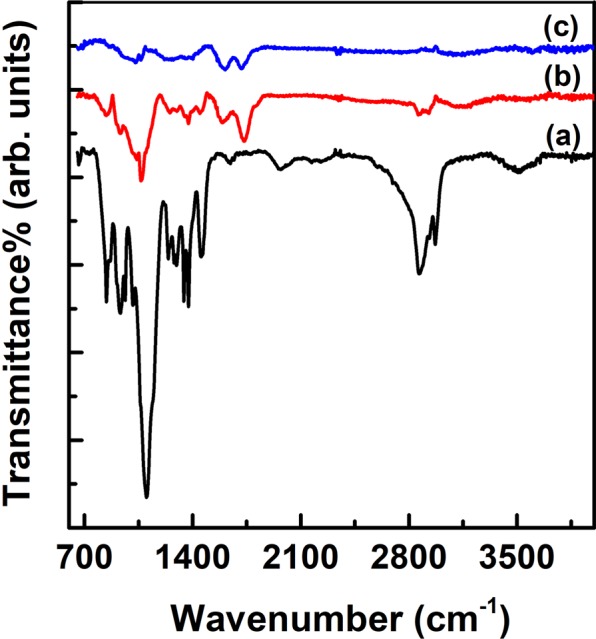
FT-IR spectra of (**a**) P123 (**b**) GO, and (**c**) SGT.

Figure 2 shows FT-IR peaks for the P123, GO and SGT. P123 peaks [Fig. 2(a)] arises at 800~1200 cm^−1^ (–C–C–), 2850~2900 cm^−1^ (–C–H) and 3200~3600 cm^−1^ (–O–H) and GO peaks [Fig. 2(b)] at 800~1200 cm^−1^ (–C–C–), 1750 cm^−1^ (–C=C–). Whereas, the characteristic peaks of P123 and GO largely decrease or nearly disappear in SGT peaks [Fig. 2(c)], indicating that P123 interacts with GO. When GO sheets are added to a Pluronic123 (P123) solution, hydrophilic groups of P123 molecules are adsorbed on the surface of GO sheets by interacting with π-electrons of GO sheet, resulting in more hydrophobic GO sheets. Such hydrophobic GO sheets form self-assembled GO aggregates, which form a roll stacked with GO sheets owing to the neutral interaction between the surface alkyl groups. In particular, the absence of –OH peak in SGT indicates that OH groups of P123 interact with π-electrons of GO sheet.

**Figure 3:**
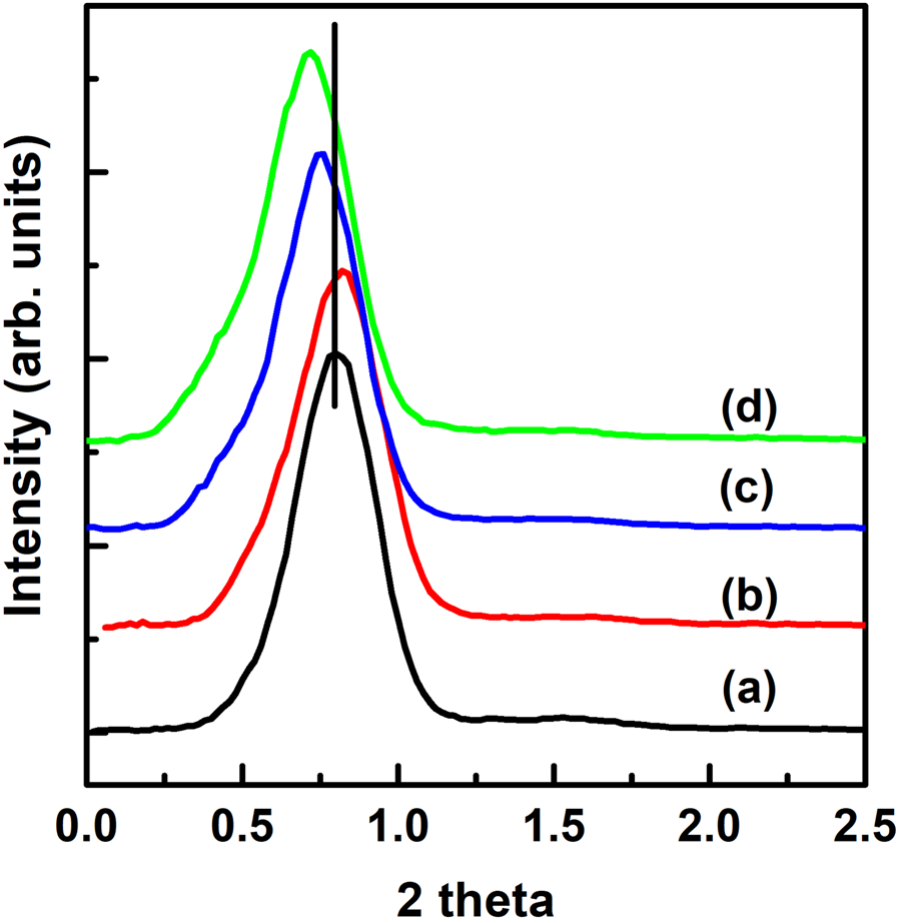
XRD patterns for as-synthesized samples of (**a**) PMS-0, (**b**) SGT-PMS-1.5, (**c**) SGT-PMS-2.2, and (**d**) SGT-PMS-3.0.

**Figure 4:**
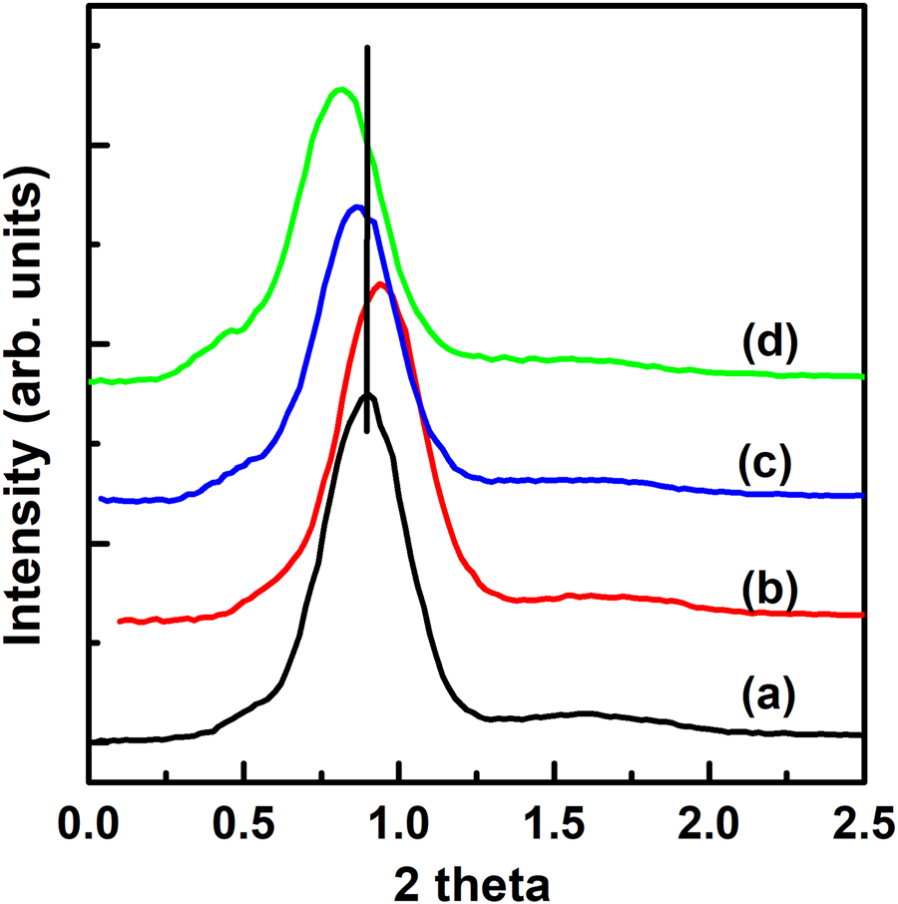
XRD patterns of (**a**) PMS-0, (**b**) SGT-PMS-1.5, (**c**) SGT-PMS-2.2, and (**d**) SGT-PMS-3.0.

**Figure 5:**
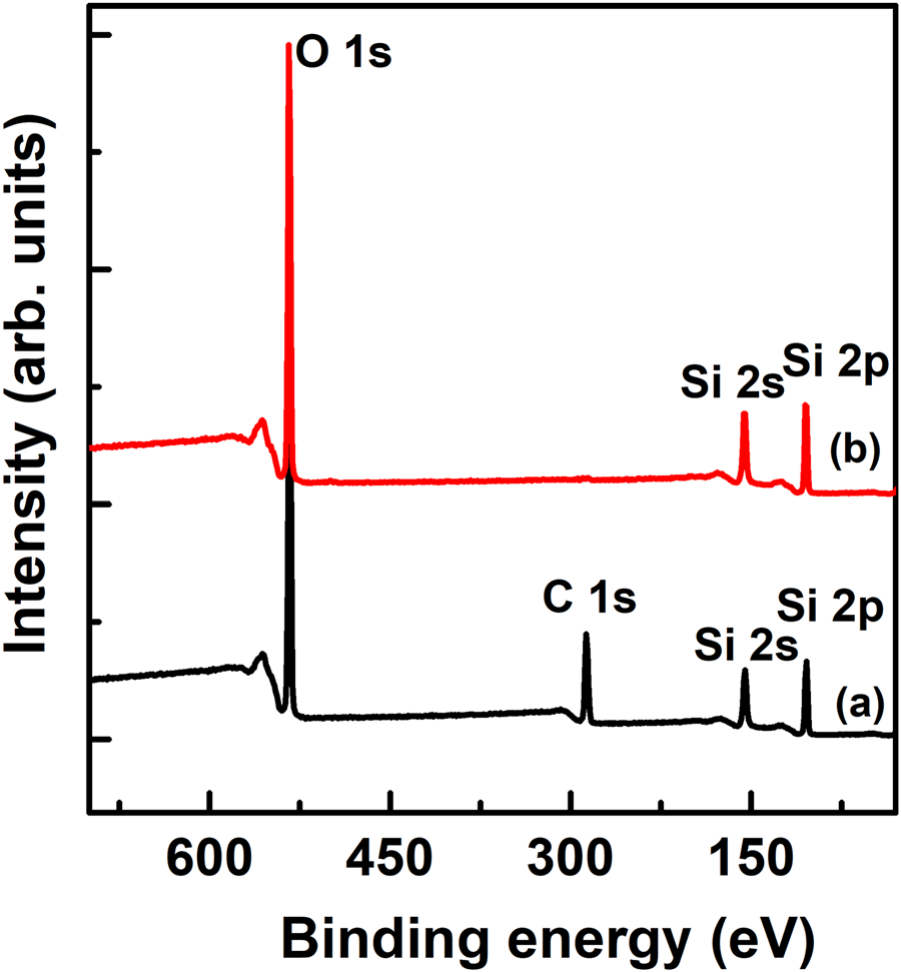
XPS spectra of (**a**) as-synthesized SGT-PMS-3.0, and (**b**) SGT-PMS-3.

**Figure 6:**
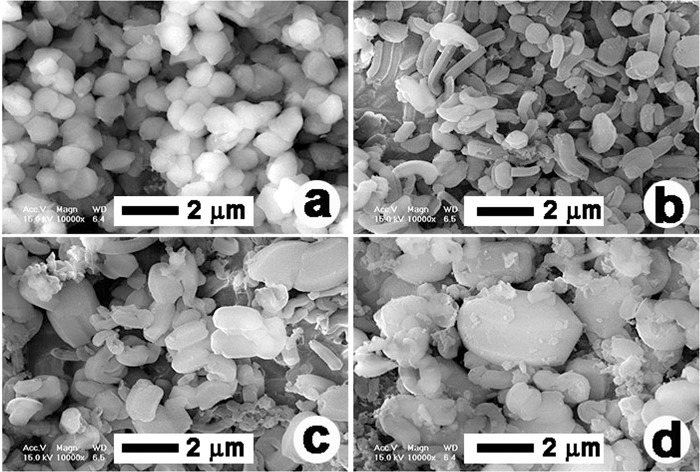
SEM images of (**a**) PMS-0, (**b**) SGT-PMS-1.5, (**c**) SGT-PMS-2.2, and (**d**) SGT-PMS-3.0.

Figure 3 shows the XRD peaks for as-synthesized PMS-0 and SGT-PMS-x(x = 1.5, 2.2, 3.0), respectively. The d-spacings of PMS-0, SGT-PMS-1.5, SGT-PMS-2.2, and SGT-PMS-3.0 were 11.35, 10.82, 11.95, and 12.30 nm, respectively. However, as shown in Fig. 4(a–d), the removal of the GO and P123 templates by calcination resulted in the decrease of d-spacing; d-spacings of PMS-0, SGT-PMS-1.5, SGT-PMS-2.2, and SGT-PMS-3.0 were now 9.8, 9.4, 10.2, and 10.8 nm, respectively. This is attributed to the contraction of gallery owing to the removal of template. Figure 5 shows XPS spectra for the as-synthesized SGT-PMS-3.0 and SGT-PMS-3.0. The absence of carbon peak indicates that organic templates such as GO and P123 are removed completely by the calcination. Figure 6 shows SEM images of PMS-0 and SGT-PMS-x(x = 1.5, 2.2, 3.0). PMS-0 shows a regular particle spherical morphology [Fig. 6(a)]. However, SGT-PMS-x samples have particle morphologies different from that of PMS-0, exhibiting irregular and elliptical large particles with various sizes and shapes [Fig. 6(b–d)]. Here, small spherical particles [Fig. 6(b)], which are caused by the PMS-0 phase, disappeared on increasing the amount of GO added [Fig. 6(d)]. This is attributed to the decrease in the P123 micelles owing to the adsorption of P123 molecules on the GO surface in accordance with the increase in the amount of added GO.

**Figure 7:**
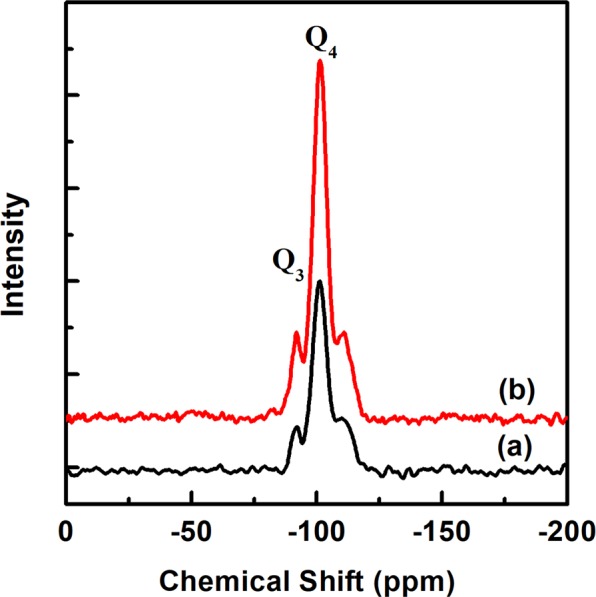
Solid-state ^29^Si MAS-NMR spectra of (**a**) PMS-0, and (**d**) SGT-PMS-3.0.

The solid-state ^29^Si MAS-NMR spectra of PMS-0 and SGT-PMS-3.0, as shown in Fig. 7(a,b), indicate that the pore wall structure of SGT-PMS-3.0 is fairly different from that of PMS-0. Here, SGT-PMS-3.0 is shown as a representative of SGT-PMS-x. In general, Si atoms of the silicate network exhibit signals (Q^2^, Q^3^, and Q^4^) in the range of ~ 90 to 115 ppm. Q^2^ (HO)_2_Si(OSi)_2_ (near 90 ppm), Q^3^ HOSi(OSi)_3_ (near 100 ppm) and Q^4^ Si(OSi)_4_ (110 to 1154 ppm) signals were observed due to the diverse environments of silicon. In particular, the intensity of Q^3^ and Q^4^ signals in the SGT-PMS-3.0 is more than double that of those for PMS-0. As the Q^3^ and Q^4^ signals are highly reflective of the degree of the silicate network development, this indicates that the pore walls in SGT-PMS-3.0 are well developed and firmer and thicker than those in PMS-0.

**Figure 8:**
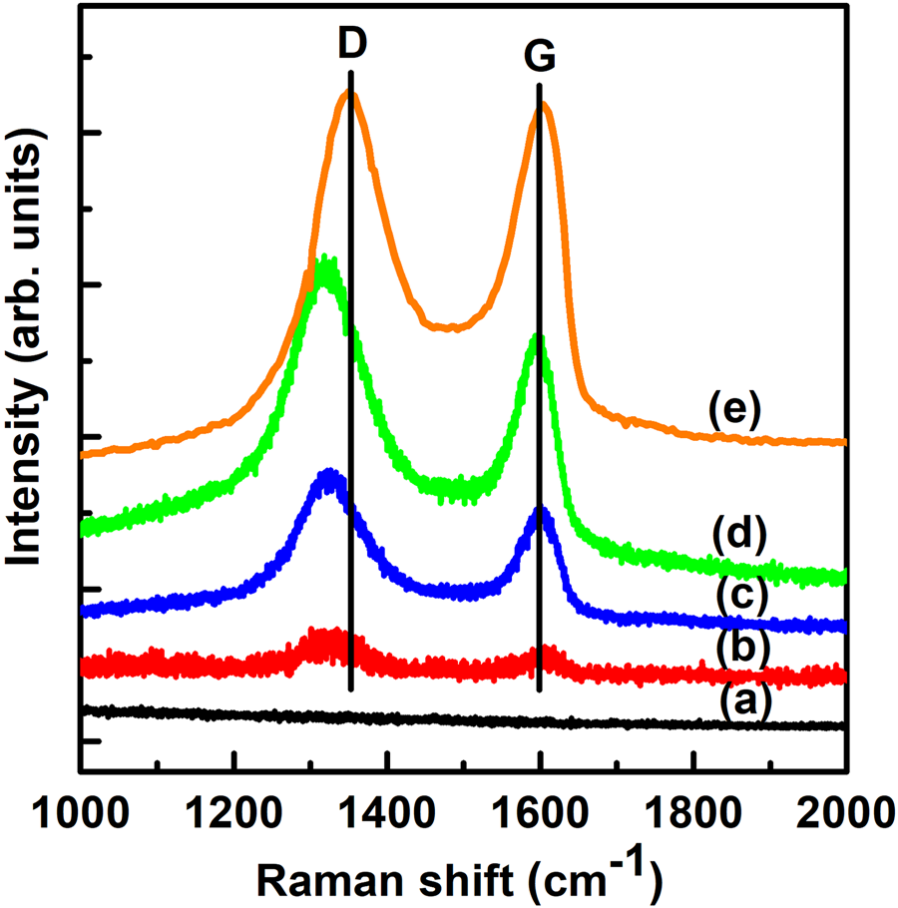
Raman spectra for as-synthesized samples of (**a**) PMS-0, (**b**) SGT-PMS-1.5, (**c**) SGT-PMS-2.2, and (**d**) SGT-PMS-3.0.

**Figure 9:**
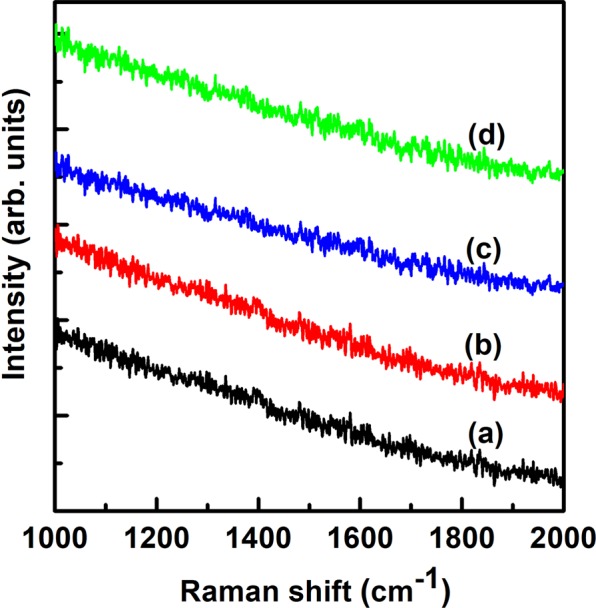
Raman spectra of (**a**) PMS-0, (**b**) SGT-PMS-1.5, (**c**) SGT-PMS-2.2, and (**d**) SGT-PMS-3.0.

Figure 8 shows the Raman spectra of as-synthesized PMS-0 and SGT-PMS-x samples. Raman spectroscopy is employed to characterize the bonding, ordering, and crystallite size in carbon materials. A Raman band arises at ~1582 cm^−1^ (the G-band) from the in-plane phonon modes of graphene, indicating *sp*^2^ bonding. The D-band at ~1353 cm^−1^ corresponds to disorder in the graphene layers caused by the presence of *sp*^3^ bonding^47^. The peaks of GO observed at ~1350 and ~1590 cm^−1^ correspond to the D and G bands, respectively^48^. However, in SGT-PMS-x, the D band occurs at 1315 cm^−1^, which is attributed to the influence of P123 adsorbed on GO surface. However, the G and D bands disappeared after calcination (Fig. 9), indicating that the GO and P123 templates were removed during calcination. XPS peaks (Fig. 5) show that the GO and P123 templates are removed during calcination.

**Figure 10:**
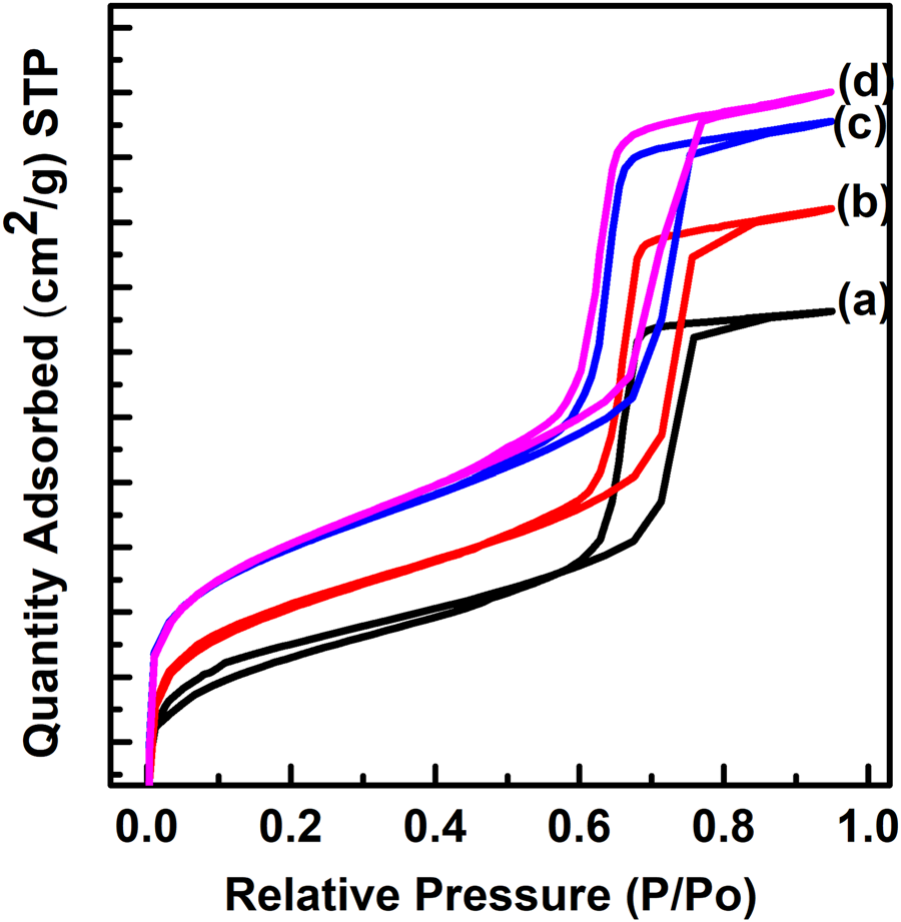
Nitrogen adsorption isotherms of (**a**) PMS-0, (**b**) SGT-PMS-1.5, (**c**) SGT-PMS-2.2, and (**d**) SGT-PMS-3.0.

**Figure 11:**
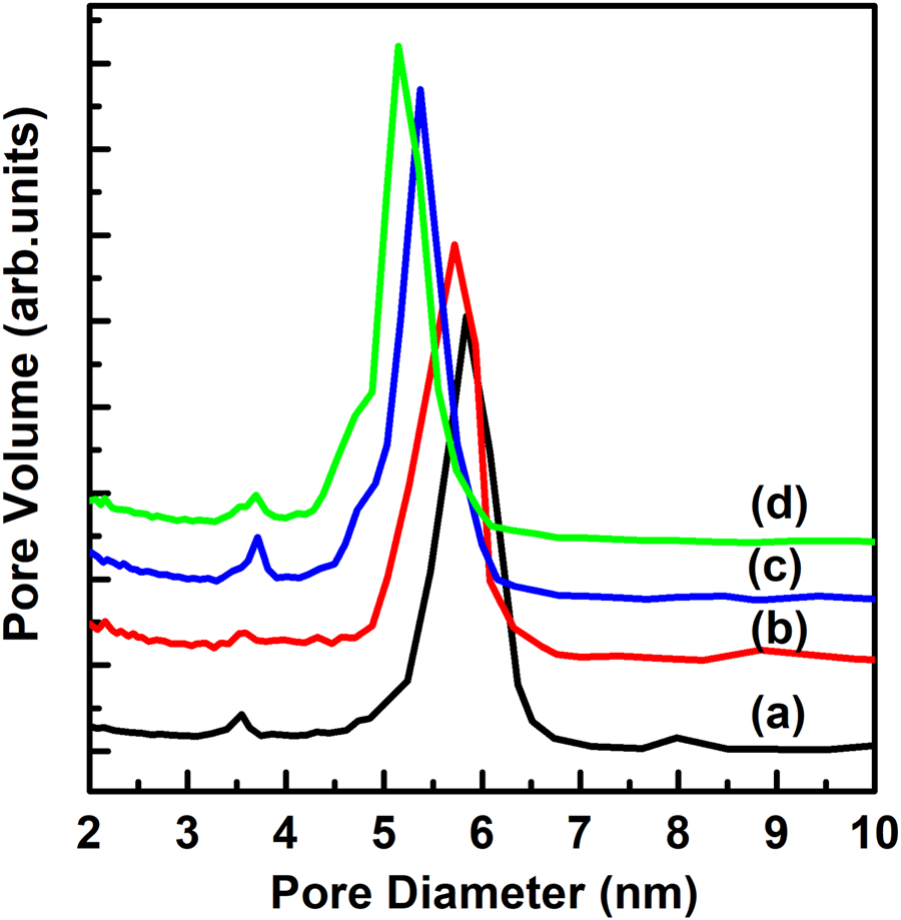
Pore size distribution obtained from the BJH adsorption isotherms of (**a**) PMS-0, (**b**) SGT-PMS-1.5, (**c**) SGT-PMS-2.2, and (**d**) SGT-PMS-3.0.

Figure 10 shows the N_2_ adsorption isotherms of PMS-0 and SGT-PMS-x samples. A typical steep increase with mesopore filling at a relative pressure of 0.6–0.8 is observed. The specific surface areas obtained using the Brunauer–Emmett–Teller (BET) equation are listed in Table 2, with considerably different values of 603.8, 713.6, 861.2, and 840.8 m^2^ g^−1^ for PMS-0, SGT-PMS-1.5, SGT-PMS-2.2, and SGT-PMS-3.0, respectively. The relatively high specific surface areas of PMS-0 and SGT-PMS-x samples indicate that PMS-0 and SGT-PMS-x are silica microparticles with microporous and mesoporous structures. In particular, the increase in surface area with the addition of GO is attributed to the increase in micropores due to the development of a pore wall. In porous materials formed using several templates, specific surface area consists of micropores developed due to the pore walls and mesopores formed by burn-off of templates. Figure 11 shows the BJH pore-size distribution of PMS-0 and SGT-PMS-x samples; pore size of PMS-0, SGT-PMS-1.5, SGT-PMS-2.2 and SGT-PMS-3.0 is 5.8, 5.6, 5.3, and 5.1 nm, respectively (Table 2). This is against the trend of *d*-spacing. In general, an increase in d-spacing results in an increase of pore size. However, if silicate sheets are thickened further by gelation using SGT, gallery height can be reduced relatively (because the gallery height = *d*-spacing - thickness of silica sheet), resulting in the decrease in pore size accompanied by the increase in surface area. This was already explained by the increase of the intensities of Q^3^ and Q^4^ peaks with the addition of GO. The increase of silica sheet thickness and the decrease of gallery height can bring out the increase of surface area, allowing the development of micropores. Therefore, the increase in surface area accompanied by the decrease of pore size are attributed to the increase in silica sheet thickness by the formation of SGT-PMS.

**Table 2:** Physical properties of PMS-0 and SGT-PMS-x.

Sample	*d*_100_ (nm)	BET surface area (m^2^ g^−1^) S_total_ S_mic_ S_meso_			Pore volume (cm^3^ g^−1^)	BJH pore diameter (nm) (adsorption)
PMS-0	9.82	603.8	139.3	464.3	0.8	5.8
SGT-PMS-1.5	9.36	713.5	190.4	523	0.85	5.6
SGT-PMS-2.2	10.24	840.8	196.5	644.2	0.96	5.3
SGT-PMS-3.0	10.83	861.2	214.5	646.5	0.99	5.1

Stotal = BET surface area.

S_micro_ = Micropore volume from t-plot; t = [14.3600/(0.1013 − lg (P/P0))]^0.5^.

S_meso_ = S_total_ − S_micro_.

**Figure 12:**
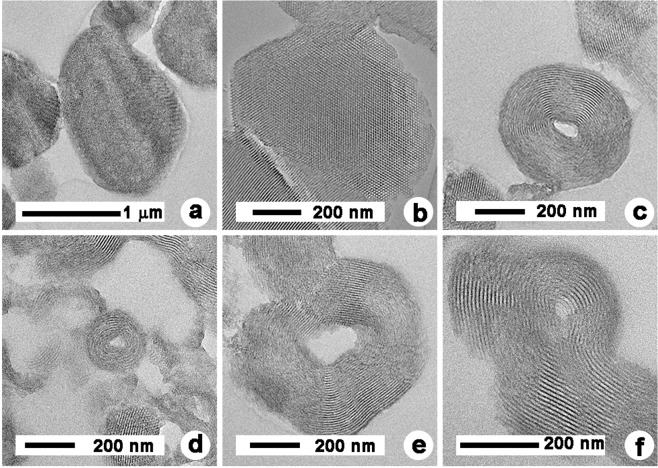
TEM images of (**a,b**) PMS-0, (**c,d**) SGT-PMS-2.2, and (**e,f**) SGT-PMS-3.0.

TEM images, as shown in Fig. 12, show the cross-section structures of SGT-PMS particles which cannot be confirmed by XRD peaks, SEM images, and ^29^Si MAS-NMR spectra. These prove that large irregular particles, as shown in SEM, are rolled in silicate sheets stacked with regular spacing. The rolls with a layered structure are also accompanied by spherical particles with nanosized hexagonal pores. Here, the nanosized hexagonal pores originated from PMS-0, whereas the rolls with layered structure originated from SGT-PMS. These results indicate that SGT-PMS is a novel mesoporous silica phase formed by SGT.

**Figure 13:**
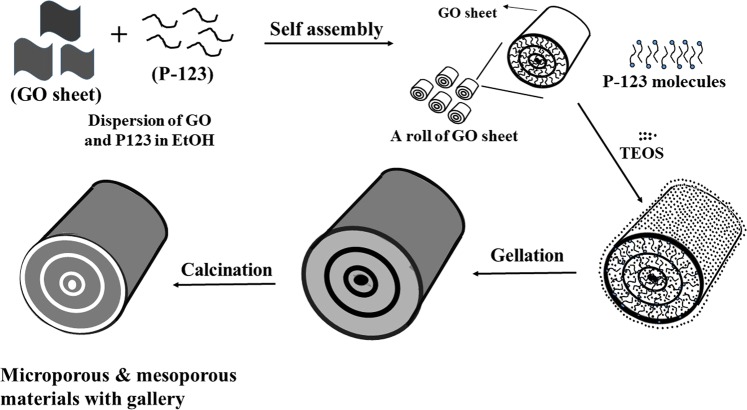
Schematic of SGT-PMS-x formed by self-assembled GO templating mechanism.

The schematic in Fig. 13 shows the SGT mechanism as a representation of the effect of the addition of GO on the physical properties such as particle morphology, regular pore structure, and surface area of SGT-PMS-x. When GO sheets are added to the P123 solution, hydrophilic head groups of P123 molecules are adsorbed on the surface of GO sheets by the molecular interaction between OH groups of P123 and π-electrons of GO sheet, resulting in hydrophobic GO sheets surrounded by alkyl chains. Such hydrophobic GO sheets form SGT like a roll stacked with GO sheets owing to neutral interaction between the alkyl chains. TEOS can be introduced into the hydrophobic galleries between GO sheets of SGT. The gelation of TEOS in the gallery of SGT results in two-dimensional thick silica sheets. The removal of the GO and P123 templates from as-synthesized samples leave only rolls comprising stacked silica sheets with regular spacing. Here, when GO is added in a small amount, SGT are accompanied by P123 micelle templates, resulting in a mixture of PMS-0 and SGT-PMS phases. The increase in the added amount of GO results in the increase in SGT-PMS-x particle size, thus leading to an increase in SGT size. Here, the galleries between silicate sheets are very regular because they are made of SGT supported by wide hydrophobic GO sheets. In future studies, we expect that mesoporous materials with various metal oxides can be produced by self-assembled GO templated gelation.

## Conclusion

Novel mesoporous silicas were prepared successfully by the gelation of TEOS using a self-assembled GO template. SGT-PMS-x showed well-developed XRD peaks with *d*-spacings of 9.8–10.8 nm depending upon the amount of GO, thereby indicating mesoporous structures. The specific surface areas were increased from 603.8 to 861.2 m^2^g^−1^, depending upon the amount of GO. The pore size distribution remained in the range 5.1–5.8 nm and the pore volume was in the range 0.80–0.99 m^3^g^−1^. However, the pore size distribution remained in the range 5.1–5.8 nm. In particular, SEM images of SGT-PMS-x showed irregular and elliptical large particles with various sizes, which are fairly different from that of PMS-0. TEM images indicate that SGT-PMS particles comprises silicate sheets with regular spacing, which are fairly different from that of PMS-0 without GO. These results were well explained by the self-assembled GO templating mechanism. The above results indicate that mesoporous materials with various metal oxides can be produced by using self-assembled GO templates.
